# Comparative multi-assay evaluation of Determine™ HIV-1/2 Ag/Ab Combo rapid diagnostic tests in acute and chronic HIV infection

**DOI:** 10.1007/s00430-019-00655-0

**Published:** 2020-02-08

**Authors:** Paul R. Wratil, Holger F. Rabenau, Josef Eberle, Marcel Stern, Maximilian Münchhoff, Imke Friedrichs, Martin Stürmer, Annemarie Berger, Susanne Kuttner-May, Dieter Münstermann, Andreas Lucht, Karolin Meixenberger, Norbert Bannert, Oliver T. Keppler

**Affiliations:** 1 Virology, National Reference Center for Retroviruses, Medical Faculty, Max von Pettenkofer Institute, LMU München, Munich, Germany; 2 German Center for Infection Research (DZIF), Partner Site Munich, Munich, Germany; 3 Institute of Medical Virology, University Hospital Frankfurt, Goethe University, Frankfurt, Germany; 4 Landeszentrum Gesundheit NRW, Bochum, Germany; 5 MVZ Labor Krone, Bad Salzuflen, Germany; 6 Division of HIV and Other Retroviruses, Robert Koch Institute, Berlin, Germany; 7 Present Address: IMD Medizinisches Versorgungszentrum, Frankfurt, Germany; 8 Present Address: Laborarztpraxis Dres. Walther, Weindel und Kollegen, Frankfurt, Germany

**Keywords:** Rapid diagnostic test, HIV, Seroconverter, Acute HIV infection

## Abstract

In resource-limited or point-of-care settings, rapid diagnostic tests (RDTs), that aim to simultaneously detect HIV antibodies and p24 capsid (p24CA) antigen with high sensitivity, can pose important alternatives to screen for early infections. We evaluated the performance of the antibody and antigen components of the old and novel version of the Determine™ HIV-1/2 Ag/Ab Combo RDTs in parallel to quantifications in a fourth-generation antigen/antibody immunoassay (4G-EIA), p24CA antigen immunoassay (p24CA-EIA), immunoblots, and nucleic acid quantification. We included plasma samples of acute, treatment-naïve HIV-1 infections (Fiebig stages I–VI, subtypes A1, B, C, F, CRF02_AG, CRF02_AE, URF) or chronic HIV-1 and HIV-2 infections. The tests’ antigen component was evaluated also for a panel of subtype B HIV-1 transmitted/founder (T/F) viruses, HIV-2 strains and HIV-2 primary isolates. Furthermore, we assessed the analytical sensitivity of the RDTs to detect p24CA using a highly purified HIV-1_NL4-3_ p24CA standard. We found that 77% of plasma samples from acutely infected, immunoblot-negative HIV-1 patients in Fiebig stages II–III were identified by the new RDT, while only 25% scored positive in the old RDT. Both RDTs reacted to all samples from chronically HIV-1-infected and acutely HIV-1-infected patients with positive immunoblots. All specimens from chronically infected HIV-2 patients scored positive in the new RDT. Of note, the sensitivity of the RDTs to detect recombinant p24CA from a subtype B virus ranged between 50 and 200 pg/mL, mirrored also by the detection of HIV-1 T/F viruses only at antigen concentrations tenfold higher than suggested by the manufacturer. The RTD failed to recognize any of the HIV-2 viruses tested. Our results indicate that the new version of the Determine™ HIV-1/2 Ag/Ab Combo displays an increased sensitivity to detect HIV-1 p24CA-positive, immunoblot-negative plasma samples compared to the precursor version. The sensitivity of 4G-EIA and p24CA-EIA to detect the major structural HIV antigen, and thus to diagnose acute infections prior to seroconversion, is still superior.

## Introduction

Effective and timely surveillance of the HIV epidemic is critical for the prevention of virus transmission and a prerequisite for achieving high rates of early antiretroviral treatment. However, approximately 17 million of the 37 million people living with HIV are unaware of their HIV status [1, 2]. Boosting and expediting the identification of infected individuals is therefore a major challenge for the global health sector in the fight against HIV/AIDS [2].

Patients suffering from acute HIV infection are particularly difficult to diagnose [3]. These individuals typically suffer from rather non-specific symptoms, yet have viral loads that are about 20 times higher than in the untreated chronic phase of infection, which also correlates with a high risk of subsequent transmissions [4, 5]. Besides that, patients with primary HIV infection will likely directly benefit from early antiretroviral therapy by improving the overall clinical outcome [6], preserving the diversity of their immune response [7, 8], possibly reducing the size of the latent HIV reservoir [9, 10], and in some cases even achieving functional cure [11]. Consequently, there is a demand for diagnostic tests that are applicable for monitoring large groups of individuals and that are capable of rapidly identifying acute HIV infections with high sensitivity and specificity.

Today, the recommended laboratory-based screening tests are fourth-generation antigen/antibody immunoassays (4G-EIAs) [12, 13]. Because of their ability to simultaneously detect anti-HIV-1/2 antibodies (IgM, IgG) and the major structural HIV-1 protein, HIV-1 p24CA, with high sensitivity, 4G-EIAs are the current gold standard to screen for chronic as well as acute HIV infections. The efficiency of diagnostic tests to identify early acute virus infections is largely determined by their ability to detect HIV-1 p24CA. The limit of detection for HIV-1 p24CA in 4G-EIAs is below 20 pg/mL and thus comparable to those of standard p24CA-EIAs [14, 15]. 4G-EIAs generally score positive 4–7 days after the HIV-1 RNA is detectable by nucleic acid amplification in plasma [16, 17]. However, 4G-EIAs require well-equipped laboratories with trained personnel, total costs in resource-poor settings can be high or even unattainable and turn-around times may be too slow for effective patient management [18].

Consequently, over the last years, rapid diagnostic tests (RDTs) for HIV screening have been widely implemented. To perform a RDT typically takes less than 30 min even for inexperienced users and tests accept different patient samples, including serum, whole blood and/or other body fluids (urine, oral swaps). Besides their utility in developing countries, rapid tests are increasingly being used also as a consumer test for self-testing in Europe and the US [19, 20].

A concern for the widespread use of RDTs has been their diagnostic performance compared to laboratory-based assays [21]. One of the most frequently employed fourth-generation RDTs is the Determine™ HIV-1/2 Ag/Ab Combo (Alere) that works with either serum or whole blood samples. Several studies indicated a poor sensitivity for the detection of HIV-1 p24CA for the early version of this RDT [22–28], and therefore, Alere developed and released a modified version of this test. The sensitivity of this modified RDT to detect HIV-1/2 antibodies was shown to be high with low rates of false positive results [29–31]. However, its reported sensitivity for the detection of HIV p24CA antigen, the critical determinant for identifying acute HIV-1 infection, varied considerably: some studies found relatively high rates of p24CA detection (> 85%) in samples from infected individuals prior to seroconversion [29, 31, 32]. In contrast, other studies reported considerably lower p24CA detection rates (< 30%) in similar patient cohorts [30, 33]. Not surprisingly, the detection rates of the HIV antigen were particularly poor in samples from patients infected with the closely related HIV-2 [30].

The aim of this study was to evaluate the performance of the new compared to the old version of the Determine™ HIV-1/2 Combo to detect p24CA as well as anti-HIV antibodies in serum samples from patients with acute (prior to or post-seroconversion) or chronic HIV-1 and HIV-2 infection. Importantly, we compared the results obtained by these RDTs to those from multiple HIV detection assays including nucleic acid amplification (PCR), 4G-EIA and p24CA-EIA. Moreover, we evaluated the capability of the new RDT to detect p24CA from several clade B HIV-1 transmitted/founder (T/F) viruses that had been cloned using viral consensus sequences isolated from patients briefly after infection [34, 35]. The performance of the new RDT to detect capsid antigen was also tested for HIV-2 strains and HIV-2 primary isolates. Furthermore, we assessed the analytical sensitivity of the RDTs for p24CA using a well-characterized protein standard [36].

## Materials and methods

### HIV detection assays

RDT assays with novel and old versions of the Determine™ HIV-1/2 Combo (Alere) were performed according to the protocol in the package insert for frozen plasma specimens [37]. Results were read between 20 and 30 min after sample addition. By visual evaluation, positive test results were either defined as strong reactive or weak reactive. If not otherwise specified, specimens were tested in a single measurement.

For comparison, we tested the specimens in parallel with ARCHITECT HIV Ag/Ab Combo (Abbott, 4G-EIA), and INNOTEST^®^ HIV Antigen mAb (Fujirebio, p24CA-EIA). Seroconversion in the plasma samples was evaluated by the New LAV Blot I Assay (Biorad) or the INNO-LIA HIV I/II Score (Fujirebio). In accordance with the manufacturers’ protocols, immunoblot results were interpreted as “positive” when showing at least two of the major bands (gp160/gp120, gp41, and p24). If HIV-1-specific bands were observed, but the criteria for positive interpretation were not met, results were termed “indeterminate”. Viral loads in HIV-1 specimens were measured using the COBAS Ampliprep/COBAS TaqMan HIV Test v2.0 (Roche, PCR). For staging of acute HIV-1 infections based on Fiebig criteria, we evaluated the presence of HIV-1 RNA (PCR) and p24CA antigen (p24CA-EIA), as well as the results of the 4G-EIA and immunoblotting: Fiebig stage I (RNA-positive, p24CA-EIA-negative, 4G-EIA-negative, immunoblot-negative), Fiebig stages II–III (RNA-positive, p24CA-EIA-positive, 4G-EIA-positive or negative, immunoblot-negative), Fiebig stage IV (RNA-positive, p24CA-EIA-positive or negative, 4G-EIA-positive, immunoblot-indeterminate), Fiebig stage V (RNA-positive, p24CA-EIA-negative or positive, 4G-EIA-positive, immunoblot-positive without p31 band), Fiebig stage VI (RNA-positive, p24CA-EIA-negative or positive, 4G-EIA-positive, immunoblot-positive with p31 band) [16, 38]. We determined the HIV-1 subtypes in positive samples by Sanger sequencing of specific parts of the *pol/rt* gene [39]. The NCBI HIV subtyping tool was used for data analysis (https://www.ncbi.nlm.nih.gov/projects/genotyping/formpagex.cgi). HIV-2 groups were identified by Sanger sequencing of specific parts of the *pol/rt*, *pol/int* and *env* genes. Obtained sequences were typed utilizing HIVAlign (Los Alamos National Laboratory sequencing database: https://www.hiv.lanl.gov/content/sequence/HIV/mainpage.html) and by applying COMET-HIV-2 (Luxembourg Institute of Health: https://comet.lih.lu/index.php?cat=hiv2) [40].

### HIV specimens

All patient samples were remnants of specimens originally submitted for HIV testing. The testing panel included 23 acutely infected, treatment-naïve, immunoblot-negative specimens (Fiebig stages I–III), 27 specimens from acutely HIV-1-infected, treatment-naïve, recently seroconverted patients (Fiebig stages IV–VI), as well as 7 samples from chronically HIV-1-infected, ART-treated patients, and 11 specimens from chronically HIV-2-infected, ART-treated patients. Furthermore, five specimens from uninfected individuals were included in the study.

### HIV-1 transmitted/founder viruses

Proviral plasmids of the following T/F viruses from subtype B were obtained from the NIH AIDS Reagent Program: WITO, CH040, CH058, CH077, CH106, RHPA, THRO, TRJO [34]. To generate T/F virus stocks, 150-mm cell culture dishes were seeded with 8 × 10^6^ HEK293T cells per plate. Subsequently, cells were transiently transfected with proviral plasmids using the transfection reagent polyethylenimine (PolySciences). Culture supernatants were harvested 48 h following transfection and passed through 0.45 µM polyvinylidene fluoride filter units (Stericup^®^, Merck). For concentrating the virions, 28 mL of harvested cell culture supernatant was layered onto 6 mL of a 25% (wt/vol) sucrose solution (prepared in phosphate-buffered saline, PBS) in a 35 ml ultracentrifuge tube, and spun down for 1.5 h at 100,000×*g* and 4 °C. After discarding the supernatants, virus pellets were re-suspended in 100 µL PBS, and stored at − 80 °C until use. HIV-1 reverse transcriptase (RT) activity was assessed in the T/F virus stocks by SG-PERT assay [41]. In addition, the infectivity of stocks was determined by the TZM-bl X-gal staining assay [42, 43]: TZM-bl cells were seeded into the wells of a 96-well plate (seeding density: 5 × 10^3^ cells/well) and subsequently treated with varying concentrations of the T/F virus stocks. 48 h post-infection, the supernatant was removed and cells were fixed with 4% paraformaldehyde in PBS for 10 min at room temperature. Afterwards, cells were stained in PBS-containing 200 μg/mL X-gal (5-bromo-4-chloro-3-indolyl-β-D-galactopyranoside, Fermentas), 1 mM MgCl_2_and 3 mM potassium ferricyanide for 4 h at 37 °C. Stained colonies were then visualized and counted under a microscope.

### HIV-2 strains and primary isolates

Heat-inactivated HIV-2 strains and primary isolates were obtained from the “Stammsammlung” of the German National Reference Center for Retroviruses (http://www.mvp.uni-muenchen.de/nationales-referenzzentrum-fuer-retroviren/hiv-stammsammlung). If the viral load in these samples was above 1 × 10^6^ c/mL, they were diluted with PBS to a calculated viral load of 1 × 10^6^ c/mL prior to testing in the RDT assays.

### HIV-1 p24CA standard

We evaluated the sensitivity of the RDTs for an HIV-1 p24CA protein standard kindly provided by Hans-Georg Kräusslich that had been bacterially expressed and purified in principle as reported [36]. The quantity of this standard was calibrated against bovine serum albumin using ultrasensitive silver staining. HIV-1 p24CA protein dissolved in the assay sample buffer of the INNOTEST^®^ HIV Antigen mAb assay was prepared at different concentrations. 50 µL of the p24CA antigen dilutions was added to the novel and old versions of the Determine™ HIV-1/2 Combo (Alere). In parallel, we examined the protein dilutions via the INNOTEST^®^ HIV Antigen mAb (Fujirebio Europe, p24CA antigen assay). Dilutions of the p24CA stock solution were prepared twice from different aliquots. The first protein preparation was tested in a single measurement. The second preparation was measured in duplicate for the RDTs and in triplicate for the p24CA-EIA (technical replicates).

### Data analysis

The performance of the novel and old versions of the RDT to detect HIV-1 p24CA and HIV-1-specific antibodies was compared in each HIV-1 specimen group (1) acutely infected, immunoblot-negative (Fiebig stages I–III); (2) acutely infected, immunoblot-indeterminate/positive (Fiebig stages IV–VI); (3) chronically infected; (4) uninfected individuals with the results from PCR, 4G-IA, and p24CA-EIA. In case of 4G-EIA, the CMIA index was calculated. A sample cutoff > 1 was defined as positive. For p24CA-EIA, the p24CA concentrations in the specimens were estimated according to the protocol provided by the manufacturer. We compared the results from testing the new version of the RDT assay on HIV-1 T/F virus samples with the results from p24CA-EIA, SG-PERT and TZM-bl X-gal staining assay. For quantification of SG-PERT, RT activities of defined dilutions of a pCHIV 528 virus suspension were used for comparison. The threshold for detecting HIV-1 p24CA of the RDT was characterized by serial dilution of the well-characterized protein standard. As a control, dilutions of the p24CA stock solution were tested in parallel by p24CA-EIA. Arithmetic means and standard deviations from this assay were calculated using Prism 7 (GraphPad Software, Inc.).

Pearson correlations with two-tailed *p* values and 95% confidence intervals were calculated via Prism 7. Specimens with viral loads > 10,000,000 c/mL were excluded when calculating correlations.

## Results

### Assay performance in acutely HIV-1-infected, treatment-naïve, immunoblot-negative specimens (Fiebig stages I–III)

The novel version of the Determine™ HIV-1/2 Combo was reactive for 77.3% (17/22) of the specimens from acutely infected, treatment-naïve, immunoblot-negative patients in Fiebig stages II–III (8/22 strong reactive, Table 1). In 5/22 cases, the assay showed reactivity for both HIV-specific antibodies and p24CA. The novel RDT identified HIV antibodies, but was not reactive for p24CA antigen in 3/22 specimens. The one specimen from an acutely infected individual in Fiebig stage I was not reactive. In comparison, the old version of the Determine™ HIV-1/2 Combo was reactive for only 25% of the specimens (0/16 strong reactive). In 1/16 specimen, the old RDT was reactive for HIV antibodies, but did not detect p24CA.

**Table 1:** Detection of acute infections by the old and new versions of the Alere RDT in plasma specimens from immunoblot-negative, treatment-naïve, HIV-1-infected patients (Fiebig I–III) compared to quantitative PCR, 4G-EIA, and p24 Ag assay

Fiebig stage	PCR (c/mL)	Old RDT		New RDT		4G-EIA	p24CA-EIA (pg/ml)	HIV subtype
		Antibody	p24 Ag	Antibody	p24 Ag	(CMIA index)		
I	59,100	N/A	N/A	−	−	−	−	B
II–III	117,600	−	−	−	−	10	65	B
II–III	140,000	−	−	−	−	1	72	B
II–III	315,000	N/A	N/A	(+)	(+)	7	271	B
II–III	475,000	N/A	N/A	+	−	7	256	B
II–III	630,000	–	−	(+)	(+)	10	N/A	N/A
II–III	870,000	−	(+)	−	+	21	729	N/A
II–III	1,160,000	−	−	−	−	8	315	CRF02_AG
II–III	1,300,000	−	−	(+)	(+)	16	649	B
II–III	1,400,000	−	−	−	−	8	238	B
II–III	1,750,000	(+)	−	(+)	−	9	312	B
II–III	2,240,000	N/A	N/A	−	−	25	686	B
II–III	2,570,000	N/A	N/A	−	(+)	63	792	B
II–III	3,340,000	N/A	N/A	−	+	62	838	B
II–III	4,600,000	−	−	−	(+)	20	916	N/A
II–III	4,800,000	−	(+)	(+)	−	19	935	B
II–III	5,060,000	N/A	N/A	+	+	87	882	B
II–III	7,500,000	−	−	−	(+)	86	1068	CRF01_AE
II–III	> 10,000,000	−	(+)	(+)	+	264	1007	B
II–III	> 10,000,000	−	−	−	+	155	878	B
II–III	> 10,000,000	−	−	−	+	530	806	F
II–III	> 10,000,000	−	+	−	+	421	1018	B
II–III	> 10,000,000	−	−	−	(+)	346	996	B
		4/16 positive (25%)		17/23 positive (73.9%)				

The COBAS Ampliprep/COBAS TaqMan HIV Test v2.0 (Roche, PCR), the INNOTEST^®^ HIV Antigen mAb (Fujirebio, p24CA-EIA), and the ARCHITECT HIV Ag/Ab Combo (Abbott, 4G-EIA) were performed on plasma specimens before measuring RDT reactivity. Immunoblot reactivity in the specimens was examined via the New LAV Blot I Assay (Biorad), or the INNO-LIA HIV I/II Score (Fujirebio). The HIV-1 subtype was determined by Sanger sequencing of the *pol/rt* region as described previously [39]. + strong reactive; (+) weak reactive; − non-reactive; *N/A* not acquired (The old RTD was no longer available for a subset of specimens that was tested in 2019)

In those samples for which we had determined the HIV-1 subtype, the old assay was reactive only for subtype B, whereas the novel RDT was reactive for samples containing subtypes B, C, as well as CRF01_AE, or F, but failed to identify anti-HIV antibodies as well as p24CA antigen in a patient specimen with subtype CRF02_AG (Table 1). All specimens from patients in Fiebig stages II–III were reactive when analyzed via 4G-EIA. The sample volume was not sufficient to perform the p24CA-EIA in 1/23 specimens. However, due to the fact that this particular specimen was PCR-positive, reactive in the 4G-EIA and immunoblot-negative, we classified it as Fiebig stages II–III. In case of the 4G-EIA, the CMIA index, and for the p24CA-EIA, the concentrations of p24CA present in the respective samples were calculated. Both parameters showed a positive correlation to the viral loads in the respective specimens (viral load in c/mL vs. CMIA index: *r* = 0.77; *R*^2^ = 0.60; *p* = 0.0003, viral load in c/mL vs. p24CA in pg/mL: *r* = 0.85; *R*^2^ = 0.73; *p* < 0.0001).

### Assay performance in acutely HIV-1 infected, treatment-naïve, immunoblot-positive specimens (Fiebig stages IV–VI)

The HIV-1 subtypes of 26/27 specimens in this group could be classified and belong to the subtypes A1, B, C, CRF01_AE, CRF02_AG or had a unique recombinant form (URF, Table 2). Both versions of the Determine™ HIV-1/2 Combo identified HIV-1 infection in all specimens from these acutely infected, immunoblot-positive patients (new RDT: 27/27, old RDT: 8/8). The old RDT only detected HIV-1-specific antibodies, whereas the new version of the assay also detected p24CA antigen in 10/27 (37%) of the patient samples. These p24CA reactive samples were from the Fiebig stages IV as well as V and showed higher viral copy numbers (> 1,900,000 c/mL). The new RDT was tested with a specimen of HIV-1 subtype C (Fiebig stage IV) that showed weak immunoblot bands for p24 and p31, in which it failed to detect HIV-1-specific antibodies.

**Table 2:** Detection of acute infections by the old and new versions of the Alere RDT in plasma specimens from acutely HIV-1-infected, treatment-naïve, immunoblot-positive patients (Fiebig IV–VI) compared to quantitative PCR, 4G-EIA, and p24 Ag assay

Fiebig stage	PCR (c/mL)	Old RDT		New RDT		4G-EIA	p24CA-EIA (pg/ml)	HIV subtype
		Antibody	p24 Ag	Antibody	p24 Ag	(CMIA index)		
IV	320,000	(+)	−	+	−	38	169	N/A
IV	1,080,000	(+)	−	(+)	−	15	336	B
IV	2,890,000	N/A	N/A	(+)	(+)	49	786	B
IV	3,410,000	(+)	−	+	−	33	817	B
IV	4,200,000	(+)	−	(+)	−	38	542	B
IV	5,190,000	N/A	N/A	(+)	+	120	851	B
IV	6,430,000	N/A	N/A	(+)	+	74	849	B
IV	6,720,000	N/A	N/A	(+)	+	202	858	B
IV	> 10,000,000	N/A	N/A	−	+	1109	909	C
V	11,300	N/A	N/A	+	−	38	−	B
V	31,400	(+)	−	+	−	13	−	B
V	446,000	N/A	N/A	+	−	21	118	B
V	801,000	N/A	N/A	(+)	−	12	299	B
V	845,000	N/A	N/A	+	−	46	317	A1
V	1,010,000	N/A	N/A	+	−	160	276	URF
V	1,200,000	(+)	−	(+)	−	13	426	CRF02_AG
V	1,210,000	+	−	+	−	51	524	B
V	1,380,000	+	−	+	−	33	886	B
V	1,950,000	N/A	N/A	(+)	(+)	27	485	B
V	3,000,000	N/A	N/A	+	(+)	93	726	CRF01_AE
V	3,450,000	N/A	N/A	+	+	66	804	B
V	4,420,000	N/A	N/A	+	(+)	77	897	B
V	> 10,000,000	N/A	N/A	(+)	(+)	232	906	URF
VI	22,500	N/A	N/A	+	−	36	−	B
VI	24,400	N/A	N/A	+	−	49	−	B
VI	878,000	N/A	N/A	+	−	102	473	B
VI	1,690,000	N/A	N/A	+	−	93	62	URF
		8/8 positive (100%)		27/27 positive (100%)				

The COBAS Ampliprep/COBAS TaqMan HIV Test v2.0 (PCR), the INNOTEST^®^ HIV Antigen mAb (p24CA-EIA), and the ARCHITECT HIV Ag/Ab Combo (4G-EIA) were performed on plasma specimens before measuring RDT reactivity. Immunoblot reactivity in the specimens was examined via the New LAV Blot I Assay or the INNO-LIA HIV I/II Score. The HIV-1 subtype was determined by Sanger sequencing of the *pol/rt* region as described previously [39]. + strong reactive; (+) weak reactive; − non-reactive; *N/A* not acquired (The old RTD was no longer available for a subset of specimens that was tested in 2019)

As a reference, the 4G-EIA was reactive for all specimens in this group. The CMIA index correlated with the viral loads in the respective specimens (*r* = 0.54, *R*^2^ = 0.29; *p* = 0.0051). However, this correlation was weaker compared to the immunoblot-negative specimen group, likely due to a mixed detection of antibodies and p24CA by the 4G-EIA. The estimated concentrations of p24CA in these samples correlated strongly with the respective viral loads (*r* = 0.75, *R*^2^ = 0.57; *p* < 0.0001). However, three specimens with low viral loads (≤ 31,400 c/mL) were not identified as p24CA-positive.

### Assay performance in specimens from chronically infected patients and uninfected control individuals

All specimens (7/7) from patients chronically infected with either HIV-1 subtype B or CRF01_AE were strong reactive in both versions of the Alere RDT (Table 3) with only HIV-1-specific antibodies being detected. In contrast, the p24CA-EIA scored positive for 2/7 samples. The 4G-EIA was highly reactive for all specimens in this group. 100% (11/11) of the specimens from chronically HIV-2-infected patients were reactive in the new version of the RDT (10/11 with strong reactivity, Table 4). All assays evaluated in this study were non-reactive for plasma specimen from uninfected individuals (Table 5).

**Table 3:** Detection of chronic HIV-1 infection by the old and new versions of the Alere RDT in plasma specimens from HIV-1-infected patients compared to quantitative PCR, 4G-EIA, and p24 Ag assay

PCR (c/mL)	Old RDT		New RDT		4G-EIA	p24CA-EIA (pg/ml)	HIV subtype
	Antibody	p24 Ag	Antibody	p24 Ag	(CMIA index)		
3060	+	−	+	−	963	−	B
3570	+	−	+	−	993	19	B
7550	+	−	+	−	982	−	B
8030	+	−	+	−	913	−	CRF01_AE
26,600	+	−	+	−	790	−	B
131,000	+	−	+	−	969	−	B
1260,000	+	−	+	−	188	48	B
	7/7 positive (100%)		7/7 positive (100%)				

The COBAS Ampliprep/COBAS TaqMan HIV Test v2.0 (PCR), the INNOTEST^®^ HIV Antigen mAb (p24CA-EIA), and the ARCHITECT HIV Ag/Ab Combo (4G-EIA) were performed on plasma specimens before measuring RDT reactivity. Immunoblot reactivity in the specimens was examined via the New LAV Blot I Assay or the INNO-LIA HIV I/II Score. The HIV-1 subtype was determined by Sanger sequencing of the *pol/rt* region as described previously [39]. + strong reactive; (+) weak reactive; − non-reactive; *N/A* not acquired

**Table 4:** Detection of chronic HIV-2 infection in PCR-reactive plasma specimens by the new version of the Alere RDT

New RDT		HIV-2 group
Antibody	Ag	
+	−	A
+	−	A
+	−	A
+	−	A
+	−	A
+	−	A
(+)	−	A
+	−	A
+	−	A
+	−	B
+	−	N/A
11/11 positive (100%)		

HIV-2 groups were determined by Sanger sequencing of specific parts of the *pol/rt*, *pol/int* and *env* genes. +  strong reactive; (+) weak reactive; − non-reactive; *N/A* not acquired. The corresponding HIV-2 virus loads were all < 2000 c/mL

**Table 5:** Specificity validation of the old and new versions of the Alere RDT, quantitative PCR, 4G-EIA, and p24CA-EIA using plasma specimens from HIV-negative individuals

PCR	Old RDT		New RDT		4G-EIA	p24CA-EIA
(c/mL)	Antibody	p24 Ag	Antibody	p24 Ag	(CMIA)	(pg/ml)
−	−	−	−	−	−	−
−	−	−	−	−	−	−
−	−	−	−	−	−	−
−	−	−	−	−	−	−
−	−	−	−	−	−	−
	5/5 negative		5/5 negative			

The COBAS Ampliprep/COBAS TaqMan HIV Test v2.0 (PCR), the INNOTEST^®^ HIV Antigen mAb (p24CA-EIA), and the ARCHITECT HIV Ag*/*Ab Combo (4G-EIA) were performed on plasma specimens before measuring RDT reactivity. Immunoblot reactivity in the specimens was examined via the New LAV Blot I Assay. − non-reactive

### Analytical sensitivity of the RDTs to detect p24CA

**Fig. 1:**
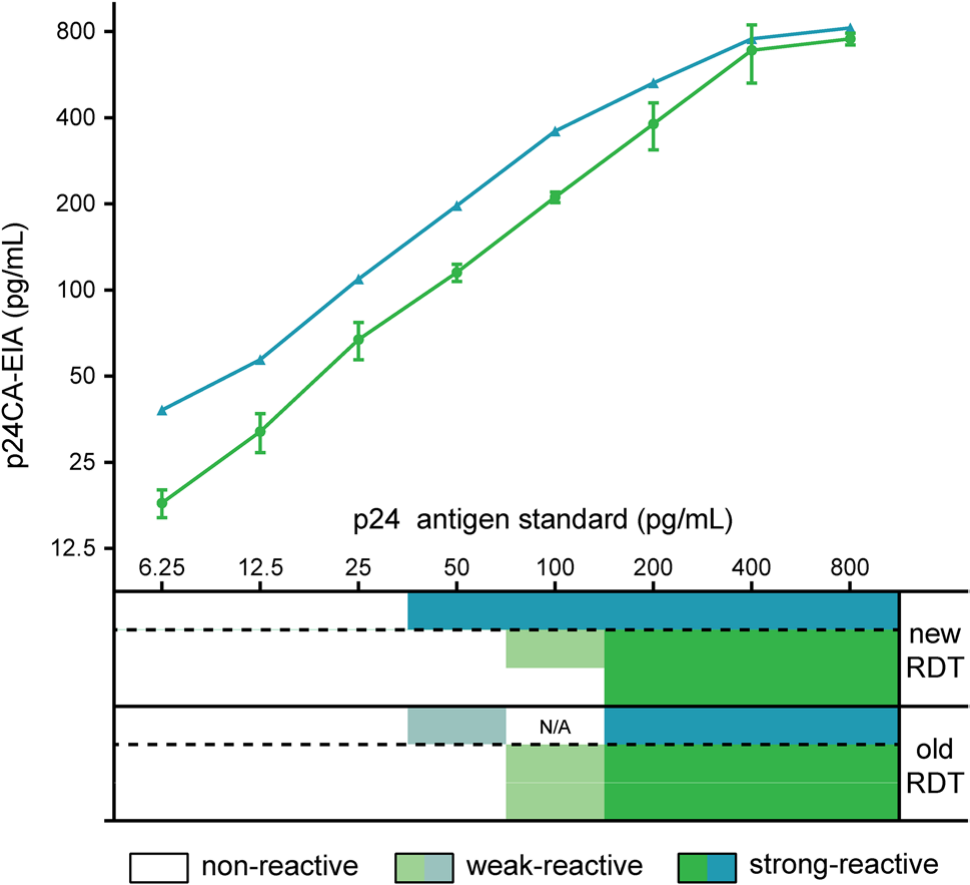
Comparative sensitivity evaluation of the old and new versions of the Alere RDT and p24CA-EIA to detect p24CA antigen. A well-characterized p24CA protein standard was used to assess the analytical sensitivity of the RDTs [32]. In parallel, reactivity of the samples was measured using the INNOTEST^®^ HIV Antigen mAb (p24CA-EIA). Two independent sets of dilutions of the highly purified p24CA stock were prepared and run in two independent assays. The first assay constituted a single measurement (results shown in blue). The second assay was run in duplicate for the RDTs and in triplicate for the p24CA-EIA (technical replicates, results shown in green, depicted are means and SD). *N/A* not acquired

We used a well-characterized p24CA antigen standard to evaluate the analytical sensitivity of the two versions of the Alere RDT to detect p24 antigen and compared the performance of this assay to the INNOTEST^®^ HIV Antigen mAb (Fujirebio, p24CA-EIA) [36]. In the first assay run in single measurement, both versions of the Determine™ HIV-1/2 Combo showed a reactivity to p24CA of ≥ 50 pg/mL (Fig. 1). In the second assay, run in duplicates, the old version of the assay was reactive only at p24CA concentrations of ≥ 100 pg/mL, whereas the novel RDT in one case had a sensitivity of ≥ 100 pg/mL, in the other ≥ 200 pg/mL p24CA. Of note, in none of the experiments these RTDs showed reactivity at 25 pg/mL, which is the p24CA sensitivity threshold in serum samples stated by the manufacturer [37]. All dilutions of the p24CA standard were in parallel subjected to testing in the p24CA-EIA. This assay showed a sensitivity of p24CA of ≥ 6.25 pg/mL. We calculated the amount of p24CA antigen detected by the test according to the manufacturer’s recommendations. The actual p24CA concentrations determined by the p24CA-EIA correlated strongly with the calculated input p24CA of the dilutions (first experiment: *r* = 0.90; *R*^2^ = 0.81; *p* = 0.0023, second experiment: *r* = 0.93; *R*^2^ = 0.87; *p* = 0.0007).

### Assay performance for HIV-1 transmitted/founder viruses

The new version of the Alere RDT detected the capsid antigen of all (8/8) clade B HIV-1 T/F viruses tested at p24CA concentrations ranging from 220 to 450 pg/ml (Table 6). However, a tenfold dilution, still at or above the test’s sensitivity according to the manufacturer, rendered all samples negative. The other virological and diagnostic tests, SG-PERT, TZM-bl infectivity assay and p24CA-EIA, successfully detected virus in all samples. As a reference, cell culture supernatants from HEK293T as well as SupT1 cells were used as negative controls, and were non-reactive when applied to the new Determine™ HIV-1/2 Combo.

**Table 6:** Detection of HIV-1 transmitted founder virus strains from concentrated cell culture supernatants by the new version of the Alere RDT compared to SG-PERT, TZM-bl X-gal infectivity assay, and p24 Ag test

Virus strain	SG-PERT* (RT U/mL)	X-gal assay* (IU/mL)	p24 Ag test (pg/ml)	New RDT (unconc.)		New RDT (10 × conc.)	
				Antibody	p24 Ag	Antibody	p24 Ag
WITO	4,615,514	2	43	−	−	−	+
CH040	2,916,079	44	22	−	−	−	(+)
CH058	2,750,968	2	26	−	−	−	+
CH077	2,546,683	17	35	−	−	−	+
CH106	5,036,899	37	40	−	−	−	+
RHPA	5,009,051	3	45	−	−	−	+
THRO	2,986,798	4	39	−	−	−	(+)
TRJO	4,322,258	3	35	−	−	−	(+)
				0/8 positive (0%)		8/8 positive (100%)	

The INNOTEST^®^ HIV Antigen mAb (Fujirebio, p24CA-EIA) as well as SG-Pert and X-gal assay were performed on the samples before measuring RDT reactivity. RDT reactivity was measured in non-concentrated samples (unconc.) and × 10 concentrated samples (× 10 conc.). The levels of p24CA antigen in the × 10 concentrated samples for RDT measurement range between 220 and 448 pg/mL, calculated based on the results of the p24CA-EIA. +  strong reactive; (+) weak reactive; − non-reactive; RT U: reverse transcriptase units; *IU* infectious units. *Shown are calculated values from measuring concentrated stocks of the same samples that were used for testing in the RDT assay: SG-PERT, TZM-bl X-gal infectivity assay (original stock: 3 × 10^6^-fold concentrated)

### Assay performance for HIV-2 viruses

HIV-2 p27 capsid antigens in samples from four HIV-2 strains and two HIV-2 primary isolates from groups A and B were not detected by the new Alere RDT (0/6 reactive, Table 7).

**Table 7:** Lack of detection of four HIV-2 strains and two HIV-2 primary isolates by the new Alere RDT compared to quantitative PCR

PCR	New RDT		Virus strain/isolate	HIV-2 group
(c/mL)	Antibody	Ag		
205,000	−	−	CBL-20	A
450,000	−	−	Rod10	A
805,000	−	−	EHO	B
1,000,000*	−	−	ATU	A
1,000,000*	−	−	MVP1845852	A
1,000,000*	−	−	MVP1847457	A
	0/6 positive (0%)			

Quantitative HIV-2 PCR was performed before measuring RDT reactivity. − non-reactive. *Samples were diluted in PBS to the calculated c/mL before performing the RDT assay

## Discussion

To our knowledge, this is the first study to analyze the performance of the novel and old RDT versions of the Determine™ HIV-1/2 Combo to detect acute (including T/F viruses) and chronic HIV infection in comparison with results from 4G-EIA, p24CA-EIA, immunoblotting, and quantitative nucleic acid amplification. Furthermore, we investigated the capabilities of the new RDT to detect chronic HIV-2 infection as well as p27CA from several HIV-2 strains and primary isolates.

The old version of the RDT reacted to only 25% (4/16) of the specimens from acutely infected, treatment-naïve, immunoblot-negative patients in Fiebig stages II–III. In only 18.8% (3/16) of the samples in this group, the old RDT identified the presence of HIV-1 p24CA. Even though the size of our cohort was limited, these data are in accordance with the results from other studies that reported poor HIV-1 p24CA sensitivity for the old RDT, ranging from 10.1 to 65.4% [22, 24, 26, 28]. Two studies conducted in sub-Saharan Africa concluded that the old version of the RDT had 0% sensitivity in specimens from acutely infected patients [44, 45]. In comparison, in our study, the novel version of the Determine™ HIV-1/2 Combo reacted to 77.3% (17/22) of the serum samples from patients in Fiebig stages II–III. This test identified the presence of p24CA in 63.6% (14/22) of the specimens from this study group. These results indicate an increased sensitivity of the new version of the RDT for detecting early HIV-1 infection prior to seroconversion. Several studies that have recently investigated the performance of the new RDT to detect infection in p24CA-positive, immunoblot-negative serum samples showed lower reactivity rates (< 30%) [30, 33], while other groups demonstrated higher reactivity rates of more than 85% in similar specimens [29, 31, 32].

All specimens from acutely HIV-1-infected, treatment-naïve, immunoblot-indeterminate/positive patients (Fiebig stages IV–VI) scored positive in both versions of the Determine™ HIV-1/2 Combo, confirming the high sensitivity of this type of RDT to detect HIV-1 infection in acute infection during or after seroconversion [29–31]. Of note, the novel RDT failed to detect HIV-1-specific antibodies in one specimen from an HIV-1 subtype C-infected patient in Fiebig stage IV. The performance of the Alere RDT to detect HIV-1 subtype C-specific antibodies in seroconverters should be studied more extensively in the future. Of note, cross-sectional data from patients with chronic HIV-1 infection in Malawi and Swaziland indicate that the Alere RDT has high sensitivity for detecting HIV-1-specific antibodies in sera from these predominantly subtype C-infected individuals [44, 45]. However, a comparison of RDT test results with immunoblot analyses and HIV-1 subtyping is missing in these studies and little is known about the performance of the RDT for detecting antibodies in immunoblot-indeterminate specimens from acutely HIV-1 subtype C-infected patients.

Of particular interest, the new version of the RDT showed HIV-1 antibody reactivity in 8/23 (34.8%) specimens from acutely infected, still immunoblot-negative patients (Fiebig stages I–III). These results indicate higher sensitivity for HIV-1-specific antibodies compared to the frequently used New LAV Blot I Assay and the INNO-LIA HIV I/II Score. This RDT may thus potentially be capable of detecting seroconversion in acute infection earlier than standard immunoblot assays, in line with recent reports [26, 46]. The old version of the RDT was reactive to HIV-specific antibodies in only 1/16 (6.3%) specimens from acutely infected seronegative patients.

Specimens from chronic HIV-1-infected patients were antibody-reactive in both versions of the Alere RDT (7/7 positive). In addition, we evaluated the performance of the novel RDT to detect chronic HIV-2 infection. Also here, the test reliably detected HIV-specific antibodies (11/11). Neither the new nor the old Determine™ HIV-1/2 Combo reacted to serum samples from uninfected individuals, underlining the already described low false positive rate of this assay [30–32].

Next, we wanted to determine the specificity of the new Alere RDT to detect p24CA antigen from clade B HIV-1 T/F viruses, originally created using viral consensus sequences from recently infected patients [34]. These strains likely resemble the genotype and phenotype of the earliest stages of HIV-1 infection better than classical laboratory strains, the latter, in specific cases, were shown to have altered capsid stability and function [47]. The capsid antigen of all T/F viruses tested (8/8) was detected by the novel RDT. However, this required p24CA concentrations ~ 10- to 20-fold higher than suggested by the test manufacturer, again raising concerns about the sensitivity of this assay component.

Due to its structural similarity to HIV-1 p24CA, HIV-2 capsid antigen can occasionally be detected by some 4G-EIAs, albeit with reduced sensitivity [48, 49]. In the current study, the new Determine™ HIV-1/2 Combo was not reactive with any of the six HIV-2 viruses tested at high viral titers, suggesting that it would be incapable of detecting acute HIV-2 infections prior to seroconversion.

The analytic sensitivity of the Determine™ HIV-1/2 Combo to detect HIV-1 p24CA antigen in serum is stated by the company to be at least 25 pg/mL [37]. In our experiments, both versions of the RDT assay failed to detect a well-characterized, highly purified p24CA antigen standard from a clade B virus strain at these concentrations [36], reaching sensitivities of only ≥ 50 pg/mL (first experiment) or even ≥ 100 pg/mL (second experiment). In one case, the novel version of the Alere RDT was reactive only at p24CA levels ≥ 200 pg/mL. In contrast, the quantitative p24CA-EIA used in this study detected the p24CA standard at concentrations ≥ 6.25 pg/mL illustrating the higher sensitivity of this type of antigen assay compared to current RDTs. Furthermore, we observed a correlation between the concentrations of p24 antigen measured by the p24CA-EIA and those that were prepared for the experiments indicating the capability of this assay to faithfully estimate the concentrations of HIV-1 p24CA antigen also in patient sera (Fig. 1). In line with the reduced sensitivity of the RDT assay for recombinant capsid antigen, detection of several clade B T/F viruses also required considerably higher p24CA antigen concentrations to score positive in the novel test version (Table 6).

The p24CA-EIA was reactive in all specimens from acutely infected, still non-seroconverted patients tested in Fiebig stages II–III. In serum samples from this study group, both versions of the Determine™ HIV-1/2 Combo were negative at p24CA concentrations ≤ 315 pg/mL. Moreover, the RDTs failed to detect the structural viral antigen also in several samples with higher p24CA concentrations (Table 1).

For acutely infected, immunoblot-indeterminate/positive specimens (Fiebig stages IV–VI), the p24CA-EIA was reactive in 23/27 (85.2%) cases. The old version of the RDT did not show reactivity to p24CA in this study group, even though 4/8 samples tested with this assay had estimated p24CA concentrations of ≥ 529 pg/mL. The new RDT detected the HIV-1 capsid antigen in 10/27 (37%) of all specimens, but all antigen-reactive specimens had estimated p24CA concentrations of ≥ 485 pg/mL. Taken together, these results strongly suggest that the sensitivity of the Determine™ HIV-1/2 Combo to detect HIV-1 p24CA antigen is considerably lower, in some cases more than 20-fold, than specified by the manufacturer [37].

The HIV-1 patient specimens evaluated in this study are mostly from subtype B, but also include the subtypes A1, C, CRF01_AE, CRF02_AG, F and URF. For future studies, it would be valuable to evaluate the performance of the RDTs with an even more diverse panel of HIV-1 specimens, especially since the old version of the RDT was shown to have impaired sensitivity to capsid antigen from non-B subtype HIV-1 [24].

Patient specimens from all HIV-1 study groups that showed positive nucleic acid amplification (except for the one Fiebig I sample) and/or positive HIV-1 immunoblot results were also reactive in the 4G-EIA, confirming that this assay has a high sensitivity to detect HIV-1 infections in plasma samples from both early acutely infected and chronically infected patients.

In samples from acutely infected patients (prior to or post-seroconversion), a larger fraction of strong reactive test results were observed using the new RDT compared to the old version of the test (Tables 1 and 2). As a consequence, positive results may be easier to spot with the new version of this rapid test, which is especially important for untrained users and self-testers.

In our study, both versions of the Determine™ HIV-1/2 Combo proved to be reactive to HIV-1-specific antibodies from several HIV-1 subtypes. Furthermore, the new RDT was reactive to antibodies from patients with HIV-2 groups A and B infection. Thus, this assay might pose a viable alternative to screen for chronic as well as acute HIV-1 and HIV-2 infections after or during seroconversion, with several advantages to other non-RDT assays, including fast turn-around times and independence from professional laboratories with trained personnel. However, even though the novel version of the RDT seems to have an improved capacity to detect also HIV-1 p24CA antigen compared to the old one, its sensitivity is markedly lower compared to p24CA-EIAs or 4G-EIAs. Moreover, based on the lack of detection of 6 HIV-2 viruses, the Determine™ HIV-1/2 Combo is likely unable to detect acute HIV-2 infection prior to seroconversion.

Taken together, the p24CA-EIA and 4G-EIA test systems should still be considered assays of choice to reliably screen for acute HIV infection. Even though the new version of the RDT shows enhanced performance, the manufacturer still needs to improve the sensitivity and likely also clade diversity of the Determine™ HIV-1/2 Combo for p24CA, to make this RDT a viable alternative to established laboratory tests for detecting acute HIV infection in immunoblot-negative patients.
